# Metabolic Disturbances Identified in Plasma Samples from ST-Segment Elevation Myocardial Infarction Patients

**DOI:** 10.1155/2019/7676189

**Published:** 2019-07-01

**Authors:** Vânia Aparecida Mendes Goulart, Anderson Kenedy Santos, Valéria Cristina Sandrim, Josimar Marques Batista, Mauro Cunha Xavier Pinto, Luiz Cláudio Cameron, Rodrigo Ribeiro Resende

**Affiliations:** ^1^Departamento de Bioquímica e Imunologia, Instituto de Ciências Biológicas, Universidade Federal de Minas Gerais, Belo Horizonte, MG, Brazil; ^2^Instituto de Ensino e Pesquisa da Santa Casa de BH, Belo Horizonte, MG, Brazil; ^3^Departamento de Química, Universidade Federal de Minas Gerais, Belo Horizonte, MG, Brazil; ^4^Departamento de Farmacologia, Instituto de Ciências Biológicas, Universidade Federal de Goiás, Goiânia, GO, Brazil; ^5^Laboratório de Bioquímica de Proteínas, Centro de Inovação de Espectrometria de Massas, Universidade Federal do Rio de Janeiro, Rio de Janeiro, RJ, Brazil

## Abstract

ST-segment elevation myocardial infarction (STEMI) is the most severe form of myocardial infarction (MI) and the main contributor to morbidity and mortality caused by MI worldwide. Frequently, STEMI is caused by complete and persistent occlusion of a coronary artery by a blood clot, which promotes heart damage. STEMI impairment triggers changes in gene transcription, protein expression, and metabolite concentrations, which grants a biosignature to the heart dysfunction. There is a major interest in identifying novel biomarkers that could improve the diagnosis of STEMI. In this study, the phenotypic characterization of STEMI patients (*n* = 15) and healthy individuals (*n* = 19) was performed, using a target metabolomics approach. Plasma samples were analyzed by UPLC-MS/MS (ultra-high-performance liquid chromatography-tandem mass spectrometry) and FIA-MS (MS-based flow injection analysis). The goal was to identify novel plasma biomarkers and metabolic signatures underlying STEMI. Concentrations of phosphatidylcholines, lysophosphatidylcholines, sphingomyelins, and biogenic amines were altered in STEMI patients in relation to healthy subjects. Also, after multivariate analysis, it was possible to identify alterations in the glycerophospholipids, alpha-linolenic acid, and sphingolipid metabolisms in STEMI patients.

## 1. Introduction

Myocardial infarction (MI) is a heart blood flow disruption that leads to tissue damage and cell death in the heart muscle. This pathology presents a high incidence worldwide, and it is a common cause of death and disability in humans [1]. MI has two major clinical manifestations: non-ST-segment elevation myocardial infarction (NSTEMI) and ST-segment elevation myocardial infarction (STEMI), which is the more severe form and main contributor to morbidity and mortality by MI [2–4]. STEMI results from the abrupt occlusion of an epicardial coronary artery; as a consequence, the myocardium distal to the occlusion site becomes ischemic [5, 6].

During the ischemic process, oxygen supply is interrupted, and mitochondrial oxidative phosphorylation rapidly stops, with a massive reduction of ATP production from energy metabolism. A compensatory increase in anaerobic glycolysis for ATP production leads to the accumulation of hydrogen ions and lactate, resulting in intracellular acidosis and inhibition of glycolysis, as well as mitochondrial fatty acid and residual energy metabolism. Impaired contraction with persistent electrical activity (excitation-contraction uncoupling) is developed in association with alterations in ion transport systems in the sarcolemma and organellar membranes [6–8].

In addition to the osmotic and ionic imbalance, the membrane depolarization also activates the voltage-dependent Ca^2+^ channels, raising levels of intracellular Ca^2+^. The rapid increase of intracellular calcium is due to the influx through the membrane and by the release of Ca^2+^ present in the mitochondria and in the cytoplasmic reticulum. Excessive cytosolic Ca^2+^ leads to the activation of calcium-dependent proteases, phospholipases, lipases, ATPases, and endonucleases. Activation of these enzymes alters cell function, destabilizes the structure of plasma membrane and cytoskeleton, increases lipolysis by free fatty acid metabolism, induces superoxide radical production, promotes DNA damage, and ultimately leads to cell death [9–12].

STEMI injury triggers changes in gene transcription, protein expression, and metabolite concentrations, which grant a biosignature of the heart dysfunction [13–15]. Detection of these biochemical changes has resulted in the discovery of emerging biomarkers, such as myoglobin, total creatine kinase (CK), CK-myocardial band, troponin I (cTnI), and troponin T (cTnT) [16]. However, the efficacy of these STEMI biomarkers is questionable because of the low sensitivity (35% for CK-MB and cTnI) and specificity (85 and 86% for CK-MB and cTnI, respectively) in the first 8 h after injury [15, 17].

The low sensitivity and specificity of biomarkers added to the fact that they can only be detected at least six hours after symptoms onset and that the MI diagnosis is based, besides other factors, in symptoms, that can vary individually, lay emphasis on the importance of the improvement of a biochemical diagnosis of MI [3, 5, 18].

The metabolomics approach has demonstrated great utility in the biomarker discovery field, as well as in detecting changes in biological pathways and in providing information on the mechanisms underlying various conditions, including cardiovascular diseases [19–21]. It is based on the global quantitative measurement of low molecular weight endogenous metabolites in tissues or biological fluids [22]. In this study, a target metabolomics approach was used to characterize the phenotypes of STEMI patients and healthy individuals. The overarching goal was to identify novel plasma biomarkers and metabolic signatures underlying STEMI.

## 2. Methods

### 2.1. Study Population

The study was conducted according to the Declaration of Helsinki, and its protocol was approved by the Ethics Committee in Research of Santa Casa Misericórdia of Belo Horizonte under number 064/2009. All subjects that agreed to participate prior to their inclusion in the study have signed an informed consent form. STEMI patients were screened based on the following inclusion criteria: age 40-80 years, gender-balanced, chest pain above 20 minutes, and electrocardiographic (ECG) features consistent with STEMI: coronary stenosis with minimal commitment of 70% of the arterial lumen in at least one coronary artery, based on the angiography results (Table 1). The exclusion criteria were as follows: prior history of myocardial infarction or stroke, non-ST-segment elevation myocardial infarction, or other acute coronary syndromes.

The control group was compound on 19 individuals who had never had heart disease or stroke. All participants were recruited from the Clinics Hospital of the Federal University of Minas Gerais (Belo Horizonte, MG, Brazil) and Santa Casa of BH (Belo Horizonte, MG, Brazil). The demographic characteristics of the patients and controls are shown in Table 2.

### 2.2. Sample Analysis

Blood samples were collected using tubes from the Vacuette® system, and 4 mL were stored in anticoagulant ethylenediaminetetraacetic acid (EDTA) to obtain plasma. The blood samples obtained were rapidly centrifuged at 3.000 rotations per minute (RPM) for 10 minutes to separate the plasma samples and then were distributed in several aliquots into microtubes and immediately stored at -80°C. Plasma samples (*n* = 15) were obtained up to seven hours after hospitalization.

A targeted metabolomics approach was used to analyze plasma samples from STEMI patients and controls. The sample preparation and analysis procedures were performed according to the AbsoluteIDQp180 kit (Biocrates Life Sciences AG, Innsbruck, Austria). This kit allows the measurement of metabolites by UPLC-MS/MS (ultra-high performance liquid chromatography-tandem mass spectrometry) and FIA-MS (MS-based flow injection analysis). Briefly, the samples were added to the center of a filter on the upper 96-well plate in 10 *μ*L aliquots per well and dried using a nitrogen evaporator. Subsequently, 50 *μ*L of a 5% solution of phenyl isothiocyanate was added for derivatization of the amino acids and biogenic amines. After incubation, the filter spots were dried again using the nitrogen evaporator.

The metabolites were extracted using 300 *μ*L of 5 mM ammonium acetate in methanol solution and transferred by centrifugation into the lower 96-deep well plate. From the obtained extract, 150 *μ*L was diluted with the same volume of H_2_0 and submitted to UPLC-MS/MS for amino acid and biogenic amine measurements. The remainder of the extract was diluted with 400 *μ*L of mass spectrometry running solvent for further MS analysis lipid detection. The UPLC-MS/MS system was equipped with an Acquity UPLC BEH C18 column (1.7 *μ*m, 2.1 × 50 mm) (Waters Chromatography, Dublin, Ireland) connected to Xevo TQ-S mass spectrometers (Waters Technologies, Massachusetts, USA), and the samples were analyzed in positive mode.

The identification and quantification of the metabolites were achieved using internal standards and multiple reaction monitoring (MRM) detection. FIA-MS analysis was performed using the tandem quadrupole mass spectrometer Xevo-TQ-S (Waters Technologies, Massachusetts, USA) also in positive mode. The data analysis and calculation of the metabolite concentrations analyzed by FIA (acylcarnitines, glycerophospholipids, sphingolipids, and hexoses) were automated using MetIDQ software (Biocrates Life Sciences AG, Innsbruck, Austria), an integral part of the kit that imports Waters' raw data files. The peaks obtained by UPLC (amino acids and biogenic amines) were analyzed using the Target Lynx Application Manager (Waters Technologies, Massachusetts, USA).

### 2.3. Statistical Analysis

The dataset was analyzed by univariate and multivariate methods. IBM SPSS (International Business Machines, New York, USA) software and the web server MetaboAnalyst 3.0 (http://www.metaboanalyst.ca) were used to develop the univariate analyses, specifically the Wilcoxon-Mann-Whitney test. These methods were used to identify the variables (metabolites) that presented statistically significant differences (*p* < 0.05) in concentrations between the groups studied.

For the multivariate analysis, the software SIMCA 14.0 (Umetrics, Umeå, Sweden) was used. The dataset containing the statistically significant variables were submitted to normalization by unit variance (UV) scaling and then to principal component analysis (PCA), partial least squares discriminant analysis (PLS-DA), and orthogonal projection to latent structure discriminant analysis (OPLS-DA) [23]. The unsupervised method, principal component analysis (PCA), was performed to verify the trends of separation between groups. Then, PLS-DA was performed. This classification technique finds the components or latent variables which discriminate as much as possible between two or more different groups of samples (X block), according to their maximum covariance with target classes (concentrations of metabolites) defined in the Y data block [24]. By relating a data matrix containing independent variables from samples (concentration values) to a matrix containing dependent variables (classes) for these samples, OPLS-DA can remove variations from the independent variables that are not correlated to the dependent variables and enables reducing the model complexity with preserved prediction ability [25].

The models were evaluated using the goodness-of-fit parameter (*R* ^2^) and the predictive ability parameter (*Q* ^2^). *R* ^2^ represents the proportion of variance explained by a given component in the model, whereas *Q* ^2^ is defined as the proportion of variance in the data predictable model under cross-validation [26]. The *R* ^2^ ranges from 0 to 1, with higher levels indicating more predictive accuracy, whereas *Q* ^2^ = 1 indicates perfect predictability [26, 27]. OPLS-DA models were examined to determine which variables were more responsible for any observed separation between groups.

To identify which variables were responsible for this separation, the variable influence on the projection (VIP) parameter was used to select variables that have the more significant contribution in discriminating between metabolomics profiles of ischemic stroke patients, STEMI, and controls. VIP scores indicate the importance of the variable to the whole model [28]. The independent variable evaluation contributes to predictions, and it is an important aspect in the multivariate classification models [29]. In our work, the independent variables were metabolite concentrations and the observation of the regression coefficients allowed us to identify which ones were more positively (high content) or negatively (low content) related to a predicted class. The web server MetaboAnalyst 3.0 (http://www.metaboanalyst.ca) was used for the construction of heatmaps and for the analysis of metabolic pathways.

## 3. Results

**Figure 1:**
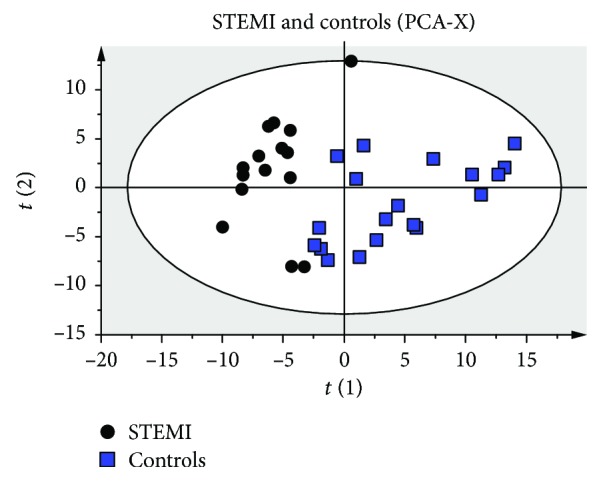
Score plots of principal component analysis (PCA) results. Score plots discriminating the metabolic profiles in plasma samples between patients with STEMI and controls. The parameters of the models were as follows: 4 PCs, *R* ^2^ = 0.679, and *Q* ^2^ = 0.47.

Concentrations of 184 metabolites were analyzed in plasma samples from STEMI patients and healthy volunteers (controls). The unsupervised PCA was first applied to explore correlations between healthy subjects and STEMI patients. According to the PCA analysis of score plots, there was a tendency towards the separation of the STEMI patients and controls (Figure 1).

**Figure 2:**
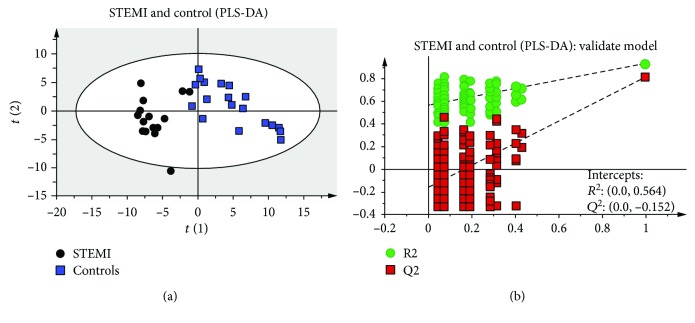
Score plots of partial least squares discriminant analysis (PLS-DA) and validation of the model. Score plots discriminating the metabolic profiles in plasma samples between patients with STEMI versus controls (a). The model's parameters were as follows: 3 latent variables, *R* ^2^ = 0.93, and *Q* ^2^ = 0.811. The plot of the permutation test of PLS-DA of STEMI versus the control group (b). Model validation with the number of permutations equaling 200.

In addition to PCA analysis, we used the same dataset for PLS-DA analyses. Permutation testing and seven-fold cross-validation, two established methods of internal validation, were used to confirm model validity. Permutation tests involve the random assignment of class labels to cases and controls [26]. The seven-fold cross-validation step involves omitting a portion of the data from model development, developing parallel models from the reduced data, predicting the omitted data from the different models, and then comparing predicted with actual values, providing an estimate of overall predictive power [30]. The obtained results demonstrated evident discrimination between the metabolic profiles (Figure 2(a)). The PLS-DA model presented high predictive and adjustment capacity (*Q* ^2^ = 0.42, *R* ^2^ = 0.93) through cross-validation. Additionally, the permutation test plot (*n* = 200) showed intercepts: *R* ^2^ = 0.0 and 0.564; *Q* ^2^ = 0.0 and 0.152, indicating that this PLS-DA model is not overfitting and is valid for this metabolomics profiling (Figure 2(b)).

**Figure 3:**
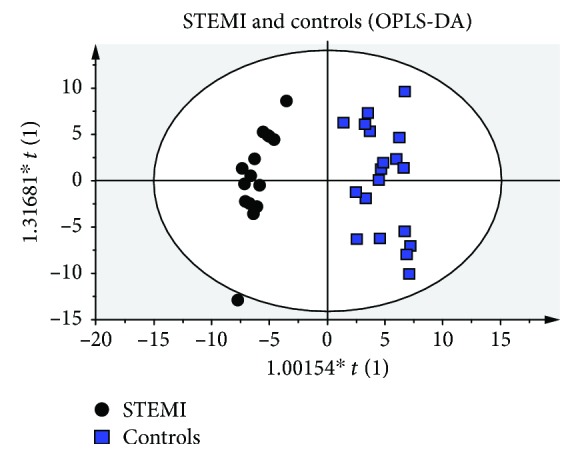
Score plots of orthogonal projection to latent structures discriminant analysis (OPLS-DA) results. Score plots discriminating the metabolic profiles in plasma samples between patients with STEMI versus controls. The parameters of the model were as follows: 2 PCs, *R* ^2^ = 0.93, and *Q* ^2^ = 0.771.

The best visualization of the discrimination between STEMI and controls was obtained through the construction of the model by OPLS-DA (Figure 3). This model, as well as PLS-DA, was validated using a seven-fold cross-validation step as internal validation. After validation, the quality parameters obtained were *R* ^2^ = 0.93 and *Q* ^2^ = 0.771.

**Figure 4:**
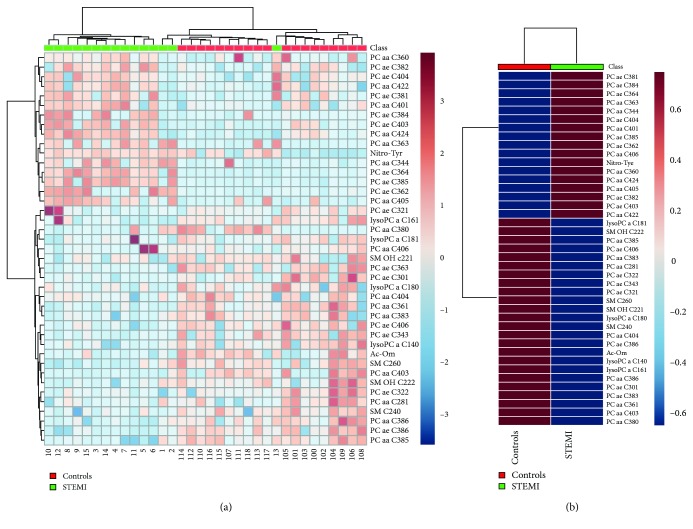
Heatmap of the metabolomics dataset. The colors represent the mean concentration of metabolites. In (a), individual samples (horizontal axis) and metabolites (vertical axis) are represented; they are separated using hierarchical clustering (Ward's algorithm), with the dendrogram being scaled to represent the distance between branches (distance measure: Euclidean). In (b), the contrast in metabolite concentrations between the patient's group with STEMI and healthy individuals is presented.

To verify which independent variables (metabolites) were more important for the classification between the groups using OPLS-DA, the VIP scores were calculated. By combining the VIP values > 1 with the results from the univariate statistical analysis, we could select the differential metabolites between STEMI patients and controls. Sixty metabolites with VIP scores > 1 were found; among them, 44 presented a statistical difference (*p* < 0.05) between STEMI and controls (Table 3). Of these metabolites, 41 had an identification record in the HMDB (Human Metabolome Database, http://www.hmdb.ca/). The differences in concentration of these metabolites among the samples are demonstrated in the heatmaps (Figure 4(a)), and the difference between groups is demonstrated in Figure 4(b) and in the graphic of concentrations (Figure 1S of the Supplementary Material). In the heatmaps, metabolites were clustered according to their Pearson correlation coefficients.

**Figure 5:**
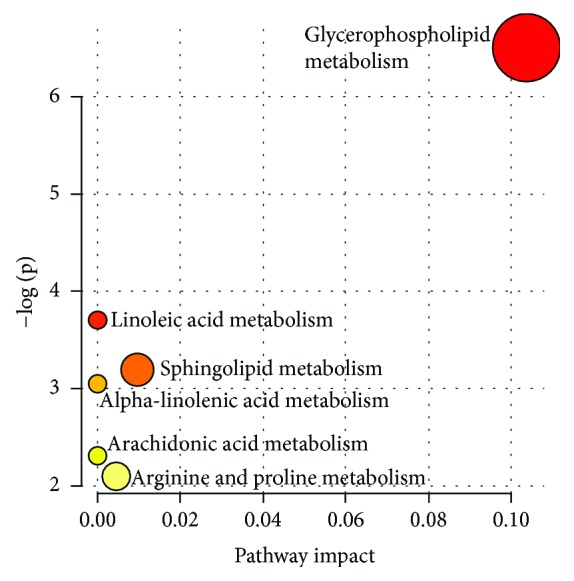
Metabolic pathways associated with STEMI. In the metabolome view, each circle represents a different pathway; circle size and color shade are based on the pathway impact and *p* value (red being the most significant), respectively.

This panel containing 41 metabolites was used to investigate the metabolic pathways associated with STEMI. Significantly altered pathways (*p* < 0.05) that also had high impact values include glycerophospholipid metabolism, alpha-linolenic acid metabolism, and sphingolipid metabolism (Figure 5). Pathway significance was determined from pathway enrichment analysis and based upon the values for each compound in the dataset. The impact value, on the other hand, was determined by pathway topology analysis. Impact represents the importance of a metabolite within a pathway; a metabolite that is found at a junction point within a pathway may have a greater impact on the pathway function if the level is altered.

The phosphatidylcholines were the main group of metabolites that showed a difference between STEMI and controls: 15 were in lower concentration and 16 in higher concentration in patients with STEMI. Four lysophosphatidylcholines and four sphingomyelins showed a lower concentration in patients with STEMI. Two biogenic amines showed a difference in STEMI: one showed high concentration and the other lower concentration (Figure 4(b)). Differences in amino acid, acylcarnitines, and hexoses concentrations between the two groups were not found.

According to the statistical model, there were no significant differences between groups related to the quantified metabolites, coronary arteries committed, and comorbidities (systemic arterial hypertension, type II diabetes mellitus, and dyslipidemia) (Tables 1 and 2). Patients with STEMI presented similar metabolic profiles, despite these variables.

## 4. Discussion

For more than three decades, the glycerophospholipid hydrolysis in a cardiomyocyte membrane during ischemia has been linked to the pathogenesis of myocardial infarction [31, 32]. The contribution of phospholipid metabolism to plasma membrane disruption in necrotic cell death induced by hypoxia or ischemia has been classically attributed to the action of phospholipases, loss of asymmetry, or the accumulation of bilayer-disrupting amphiphilic lipids, such as lysophospholipid [12]. In general, alterations in myocardial lipid metabolism during ischemia/reperfusion can be classified into two groups: (1) changes in fatty acid *β*-oxidation and (2) changes mediated by the activation of phospholipases and other lipid catabolic enzymes (e.g., ceramidase and sphingomyelinase) that target the structurally important lipid constituents of cellular membrane structures of the heart [8].

The major lipids present in the eukaryotic cell membrane are glycerophospholipids, sterols, and sphingolipids [15]. According to our results, the main metabolic pathway associated with STEMI is the metabolism of glycophospholipids, followed by alpha-linoleic acid metabolism and sphingolipid metabolism. The major classes of glycerophospholipid include phosphatidylcholine, phosphatidylethanolamine, phosphatidylserine, phosphatidylinositol, and phosphatidic acid [14]. Plasmalogens are unique phospholipid species particularly derived from phosphatidylcholine (PC) or phosphatidylethanolamine (PE); they are characterized by the presence of a vinyl-ether bond and an ester bond at the *sn*-1 and *sn*-2 positions, respectively, of the glycerol backbone [33]. On the basis of their polar head groups at the *sn*-3 position, plasmalogens are mainly classified into either choline plasmalogens or ethanolamine plasmalogens [34].

Glycerophospholipids have received special attention in research on myocardial infarction and its causes [19, 22]. Phosphatidylcholine is the principle phospholipid in the mammalian heart [21]. The human cardiomyocytes are composed of approximately 40% of phosphatidylcholines [20, 21]. Compared to the control group, we found 15 phosphatidylcholines in a lower concentration in the plasma of STEMI patients. In a cardiac tissue, previous studies reported the inhibition of phosphatidylcholine synthesis during hypoxia or ischemia. Hatch and Choy described the inhibition of phosphatidylcholine synthesis in perfused hearts undergoing hypoxia [35], phosphatidylcholine synthesis was also impaired by hypoxia in isolated rat ventricular myocytes [36], and a net loss of choline after global ischemia has been recently demonstrated in reperfused rat hearts [37]. In this context, several authors suggested that the depletion of ATP and CTP was the cause of the reduced phosphatidylcholine synthesis [35, 38, 39].

Recently, it has been shown in two apparently healthy middle-aged adult cohorts that serum concentrations of four sphingomyelins and six phosphatidylcholines were associated with a higher risk of STEMI, regardless of several risk factors for cardiovascular disease. These are PC aa C38:3, PC aa C40:4, PC ae C36:3, PC ae C38:3, PC ae C38:4, and PC ae C40:3, as well as sphingomyelins C16:0, C24:0, and C16:1 and hydroxy-sphingomyelin C22:1 [21]. According to our results, seven of these markers prevailed after STEMI, two in higher concentrations in relation to the control group (PC ae C38:4 and PC ae C40:3) and five in lower concentrations (PC aa C38:3, PC aa C40, and PC ae C36:3; sphingomyelins C24:0 and hydroxy-sphingomyelin C22:1). Some biologically active substances during ischemia, for example, tumor necrosis factor-*α* (TNF-*α*), may induce the synthesis of ceramide from sphingomyelin via sphingomyelinase. Ceramide, in turn, may act as a second messenger, promoting cardiomyocyte apoptosis [40, 41]. This may be an explanation for the decrease of sphingomyelins in STEMI patients.

Substantial evidence accumulated in the last decade indicates that glycerophospholipids, specifically plasmalogens, could represent a major lipid-soluble antioxidant component [42–44]. This proposal is based on the ability of the plasmalogens to scavenge several reactive oxygen species, their relatively high concentrations in cardiac tissue, and their subcellular and extracellular locations in close vicinity to the oxidizable substrates [34, 39]. In this way, oxidative stress can be a justification for the decrease of phosphatidylcholines (choline plasmalogens) in myocardial infarction.

On the other hand, we found 16 phosphatidylcholines in higher concentration in STEMI patients. Furthermore, orthogonal partial least squares discriminant analysis (OPLS-DA) indicated that STEMI patients can be differentiated from healthy volunteers by the phosphatidylcholine PC ae C36:4. Some hypotheses may be suggested to justify these results: the wide variety of phospholipase A and their selectivity to certain substrates [45, 46] and the activation enzyme kinetics [47]. Using Langdorf rabbits as an experimental model of global myocardial ischemia, Hazen and colleagues analyzed the action of the calcium-independent plasmalogen-selective phospholipases A_2_ enzyme. It was reported that membrane-associated calcium-independent plasmalogen-selective phospholipase A_2_ activity increased over 400% during 2 min of global ischemia, was nearly maximally activated (greater than 10-fold) after only 5 min of ischemia, and remained activated throughout the entire ischemic interval examined (2-60 min). The activation of membrane-associated plasmalogen-selective phospholipase A_2_ after 5 min of myocardial ischemia was rapidly reversible during reperfusion of ischemic tissue [8]. This example suggests that after reperfusion, past the process of injury and inflammation, phospholipid biosynthesis may increase during the repair process in the same way as it occurs with cholesterol and other biomolecules [48].

A reduction in four lysophospholipids was also observed in STEMI patients (lysoPC a C18:1, lysoPC an 18:0, lysoPC a C14:0, and lysoPC a C16:1) when compared to healthy volunteers. The results of Zhu and colleagues corroborate our findings [49]. Using the UHPLC method, they observed a reduction of three lysophospholipids (lysoPC a C18:2, lysoPC a C16:0, and lysoPC a C18:1) in serum concentration from MI patients. The difference between our works lies in the fact that their samples were collected in the period of 1-6 months after the infarction [49]. Interestingly, it can be observed that one of the potential markers found in STEMI patients in the acute phase (lysoPC an 18:1) are also present in the chronic phase of the disease.

Nitrotyrosine is produced by the modification of protein tyrosine residues by peroxynitrite generated from the reaction of nitric oxide (NO) and superoxide [50]. In our study, it was found to be increased in STEMI patients. This increase has already been reported in patients with cardiovascular pathologies, being considered a transient change during myocardial ischemia [51, 52]. Regarding acetylornithine, its decrease was reported in the early stages of cardiotoxicity induced by a potent chemotherapeutic agent, doxorubicin in male B6C3F1 mice, but its implication on human myocardial infarction is described here for the first time [53].

In this study, we did not find a significant difference between STEMI patients and controls in the metabolic classes: amino acids, acylcarnitines, and hexoses. It has been reported that the amino acid concentration increasing is related to the major risk of adverse events and death in STEMI patients after primary percutaneous coronary intervention [9, 10]. Elevated levels of acylcarnitines were associated with a higher risk of myocardial infarction and diabetes mellitus. Despite this, in this work, we did not find metabolic differences between STEMI patients with diabetes mellitus and STEMI patients without this disease, and neither did we observe alterations in hexoses concentrations. Other works with bigger samples have demonstrated that glycemic alterations can be related to worse prognosis and with a smaller capacity of myocardial regeneration/healing [54, 55].

## 5. Conclusion

In conclusion, the present study suggests that there are significant alterations in the metabolism of glycerophospholipids, alpha-linolenic acid metabolism, and sphingolipid metabolism in STEMI patients. These changes were observed in the concentrations of 31 phosphatidylcholines, four lysophosphatidylcholines, four sphingomyelins, and two biogenic amines. Although this work had a limitation in the number of samples, the results confirm trends exhibited in previous studies. In addition, this work points to metabolites with a great potential to be biomarkers for STEMI and for the study of new pharmacological targets.

## Figures and Tables

**Table 1:** The number of cases and affected arteries in patients with STEMI.

Affected arteries^∗^	Cases (%)
*One artery*	
Left anterior descending coronary artery (LAD)	5 (33.3)
Right coronary artery (RCA)	1 (6.6)
Left circumflex coronary artery (LCX)	1 (6.6)
*Two arteries*	
Right coronary artery+left anterior descending coronary artery	3 (20)
Right coronary artery+left circumflex coronary artery	3 (20)
Left anterior descending coronary artery+posterior descending artery	2 (13.3)

^∗^Information obtained through coronary angiography.

**Table 2:** Demographic data of patients and controls.

Patients		Age^∗^	SAH^∗∗^ (%)	Smoking (%)	Dyslipidemia (%)	Type 2 diabetes mellitus (%)
*STEMI*	*n* = 15					
Male	8	66.72 (±4.92)	50.0	62.5	12.5	12.5
Female	7	69.43 (±9.95)	57.2	28.5	28.5	42.8
*Controls*	*n* = 19					
Male	9	55.56 (±12.07)	44.4	44.4	—	—
Female	10	58.30 (±7.70)	70.0	30.0	—	—

^∗^Mean (±SD). ^∗∗^SAH: systemic arterial hypertension.

**Table 3:** Differentiating metabolites between STEMI and healthy individuals (controls).

No.	Metabolites	Classes	VIP^a^	FC^b^	*p* value^c^	*q* value^d^	STEMI	Controls
1	PC ae C36:4	Phosphatidylcholines	1,49	-3.69	**0.0003**	0.0049	Up	Down
2	PC ae C36:3	Phosphatidylcholines	1,48	1.98	**0.0021**	0.0140	Down	Up
3	PC ae C34:2	Phosphatidylcholines	1,47	1.05	0.8082	0.8841	Down	Up
4	PC ae C38:5	Phosphatidylcholines	1,44	-3.65	**0.0014**	0.0119	Up	Down
5	PC ae C38:6	Phosphatidylcholines	1,42	2.27	**< 0.0001**	0.0000	Down	Up
6	PC ae C34:3	Phosphatidylcholines	1,39	1.92	**0.0020**	0.0140	Down	Up
7	PC aa C34:4	Phosphatidylcholines	1,39	-2.4	**0.0076**	0.0285	Up	Down
8	PC ae C40:3	Phosphatidylcholines	1,38	-2.29	**0.0442**	0.0913	Up	Down
9	PC aa C40:2	Phosphatidylcholines	1,37	1.22	0.5324	0.6310	Down	Up
10	PC ae C40:6	Phosphatidylcholines	1,35	1.64	**0.0022**	0.0142	Down	Up
11	lysoPC a C14:0	Lysophosphatidylcholines	1,35	1.68	**0.0004**	0.0049	Down	Up
12	PC aa C36:1	Phosphatidylcholines	1,33	1.7	**< 0.0001**	0.0017	Down	Up
13	PC aa C36:3	Phosphatidylcholines	1,33	-3.29	**0.0302**	0.0729	Up	Down
14	PC ae C36:5	Phosphatidylcholines	1,32	-1.21	0.7157	0.8035	Up	Down
15	PC ae C32:1	Phosphatidylcholines	1,32	1.14	**0.0076**	0.0285	Down	Up
16	PC aa C36:0	Phosphatidylcholines	1,32	-1.44	**0.0136**	0.0426	Up	Down
17	PC aa C40:3	Phosphatidylcholines	1,30	3.0	**0.0264**	0.0663	Down	Up
18	PC ae C38:4	Phosphatidylcholines	1,29	-3.69	**0.0003**	0.0049	Up	Down
19	PC aa C38:3	Phosphatidylcholines	1,29	1.54	**0.0003**	0.0049	Down	Up
20	PC aa C42:4	Phosphatidylcholines	1,28	-1.6	**0.0045**	0.0190	Up	Down
21	SM C26:0	Sphingomyelins	1,27	2.42	**0.0002**	0.0036	Down	Up
22	lysoPC a C18:2	Lysophosphatidylcholines	1,25	1.58	**0.0045**	0.0190	Down	Up
23	PC aa C30:0	Phosphatidylcholines	1,23	1.82	**0.0004**	0.0049	Down	Up
24	PC ae C42:1	Phosphatidylcholines	1,22	-1.35	0.1401	0.2330	Up	Down
25	PC ae C38:3	Phosphatidylcholines	1,22	2.23	0.0860	0.1594	Down	Up
26	PC ae C36:2	Phosphatidylcholines	1,21	-4.02	**0.0315**	0.0733	Up	Down
27	PC ae C34:1	Phosphatidylcholines	1,20	1.05	0.5324	0.6310	Down	Up
28	Nitro-Tyr	Biogenic amines	1,20	-2.18	**0.0008**	0.0075	Up	Down
29	PC aa C38:5	Phosphatidylcholines	1,20	1.87	**<0.0001**	0.0017	Down	Up
30	SM (OH) C24:1	Sphingomyelins	1,19	-1.14	0.1760	0.2715	Down	Down
31	lysoPC a C20:3	Lysophosphatidylcholines	1,19	1.04	0.3764	0.4824	Down	Up
32	PC ae C38:1	Phosphatidylcholines	1,17	-2.11	**0.0183**	0.0533	Up	Down
33	PC ae C44:3	Phosphatidylcholines	1,16	-1.32	0.3488	0.4603	Down	Down
34	Ac-Orn	Biogenic amines	1,16	1.82	**0.0039**	0.0190	Down	Up
35	PC aa C40:5	Phosphatidylcholines	1,15	-2.44	**0.0201**	0.0548	Up	Down
36	PC ae C32:2	Phosphatidylcholines	1,15	1.55	**0.0005**	0.0052	Down	Up
37	PC ae C30:1	Phosphatidylcholines	1,15	1.98	**0.0159**	0.0474	Down	Up
38	PC ae C40:4	Phosphatidylcholines	1,14	-1.68	**0.0044**	0.0190	Down	Down
39	PC aa C40:1	Phosphatidylcholines	1,13	-1.44	**0.0376**	0.0829	Up	Down
40	SM (OH) C14:1	Sphingomyelins	1,13	1.07	0.2746	0.3779	Down	Up
41	PC ae C34:0	Phosphatidylcholines	1,13	-1.42	0.9171	0.9403	Up	Down
42	SM C24:0	Sphingomyelins	1,13	1.41	**0.0001**	0.0035	Down	Up
43	PC aa C38:4	Phosphatidylcholines	1,13	-2.04	0.1600	0.2561	Up	Down
44	lysoPC a C16:1	Phosphatidylcholines	1,12	1.59	**0.0080**	0.0293	Down	Up
45	PC aa C40:6	Phosphatidylcholines	1,11	-1.02	**0.0329**	0.0738	Up	Down
46	SM (OH) C22:1	Sphingomyelins	1,11	1.55	**0.0119**	0.0391	Down	Up
47	PC aa C38:0	Phosphatidylcholines	1,11	1.29	**0.0113**	0.0388	Down	Up
48	SM (OH) C22:2	Sphingomyelins	1,11	1.62	**0.0018**	0.0136	Down	Up
49	PC aa C42:2	Phosphatidylcholines	1,10	-1.44	**0.0201**	0.0548	Up	Down
50	PC ae C44:5	Phosphatidylcholines	1,10	-1.58	0.2382	0.3397	Up	Down
51	PC ae C44:6	Phosphatidylcholines	1,07	-1.72	0.3580	0.4675	Up	Down
52	lysoPC a C18:1	Lysophosphatidylcholines	1,07	1.27	**0.0329**	0.0738	Down	Up
53	PC aa C24:0	Phosphatidylcholines	1,07	-1.77	**0.0090**	0.0321	Up	Down
54	PC aa C38:6	Phosphatidylcholines	1,06	1.69	**0.0009**	0.0084	Down	Up
55	PC aa C28:1	Phosphatidylcholines	1,03	1.48	**0.0263**	0.0663	Down	Up
56	PC ae C30:0	Phosphatidylcholines	1,03	1.58	0.1761	0.2715	Down	Up
57	PC ae C38:2	Phosphatidylcholines	1,02	-1.81	**0.0033**	0.0184	Up	Down
58	lysoPC a C18:0	Lysophosphatidylcholines	1,01	1.21	**0.0315**	0.0733	Down	Up
59	PC aa C40:4	Phosphatidylcholines	1,01	1.41	**0.0040**	0.0190	Down	Up
60	PC aa C32:0	Phosphatidylcholines	1,01	-1.5	0.9862	0.9862	Up	Down

^a^Variable importance in the projection (VIP) was obtained from OPLS-DA with a threshold of 1.0. ^b^The fold change (FC) was calculated by the average value of the STEMI group to that of the control group. ^c^ *p* value was calculated by the Wilcoxon-Mann-Whitney test. *p* values < 0.05 are in bold. ^d^ *q* value was the adjusted *p* value with the false discovery rate (FDR).

## Data Availability

The data used to support the findings of this study are available from the corresponding authors upon request.

## Supplementary Materials

Supplementary Materials Figure 1S: mean of metabolic concentrations. All presented metabolites have a *p* value < 0.05 and VIP scores (variable influence on the projection) greater than 1.
