# Supplementary material for: Metabolic Disturbances Identified in Plasma Samples from ST-Segment Elevation Myocardial Infarction Patients
Source: Dis Markers. 2019 Jul 1;2019:7676189. doi: 10.1155/2019/7676189 (PMC6636502; doi:10.1155/2019/7676189)
Supplement: Supplementary Materials — Figure 1S: mean of metabolic concentrations. All presented metabolites have a p value < 0.05 and VIP scores (variable influence on the projection) greater than 1. [file 7676189.f1.pdf]

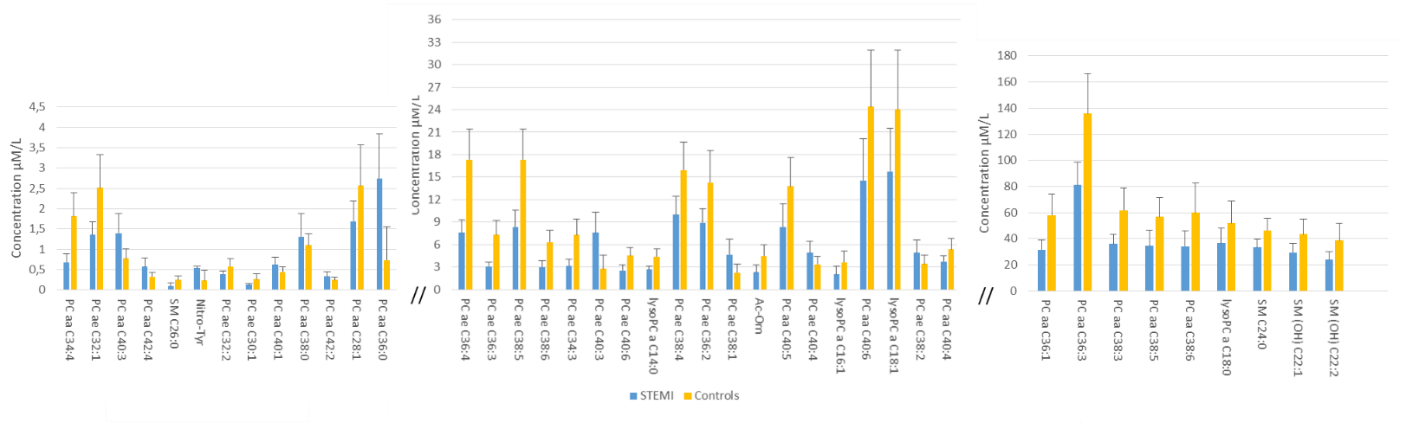

Figure 1S. Mean of metabolic concentrations. All presented metabolites have a *p*-value <0,05 and VIP scores (variable influence on the projection) greater than 1.
